# Prevalence of *Clostridium difficile* and its toxigenic genotype in beef samples in west of Iran

**Published:** 2017-06

**Authors:** Malihe Kheradmand, Somayeh Jalilian, Amirhooshang Alvandi, Ramin Abiri

**Affiliations:** Department of Microbiology, Faculty of Medicine, Kermanshah University of Medical Sciences, Kermanshah, Iran

**Keywords:** *Clostridium difficile*, Ground beef, PCR

## Abstract

**Background and Objectives:**

*Clostridium difficile* is the leading cause of nosocomial diarrhea and pseudomembranous colitis. The prevalence of *C. difficile* infection differs in various geographical areas. The aim of this study was to determine the prevalence of *C. difficile* isolates and the prevalence of *cdd3, tcdA* and *tcdB* genes in beef samples in Kermanshah Province.

**Materials and Methods:**

One hundred ground beef samples were randomly collected from the butchers of Kermanshah province during March 2014 to March 2015. Following alcohol shock, minced meat samples were incubated in a specific culture medium for 5 to 7 days. The suspicious colonies were analyzed by biochemical tests and frequency of *C. difficile* and *cdd3, tcdA* and *tcdB* genes was assessed by PCR using specific primers.

**Results:**

In total, 30% samples were positive for *C. difficile* and all the isolates harbored *Cdd3* gene. Combined dual-gene frequency of A^+^B^+^, A^−^B^+^ and A^+^B^−^ strains in the positive were 0%, 3.3%, and 26.6% respectively, while 21 samples (70%) were non-toxigenic (A^−^B^−^).

**Conclusion:**

In this study, the presence of *C. difficile* in beef as a source of contamination was confirmed. It was also shown that the incidence of *C. difficile* in ground meat was relatively higher than many other studies.

## INTRODUCTION

*Clostridium difficile* is a gram positive, spore-forming obligatory anaerobic bacterium (1). As the produced spores are highly resistant to harsh environments, *C. difficile* can sustain and live at various surfaces (2). *C. difficile* was isolated for the first time from stool of healthy newborns in 1935, while its virulence was confirmed in 1970s (3). The infection is a major global problem and is important in children and newborns. Nearly 25 to 80% of children and newborns are colonized without any symptoms and diarrhea (4) although it can cause colonic and extra-colonic infections. Colonization in the colon causes asymptomatic infection, pseudomembranous colitis, and fatal colitis with colonic perforation, peritonitis and eventually death (5). The prevalence of diarrhea associated with *C. difficile* led to a high mortality rate in Canada and America in 2003 (6). In a healthy person, the bacterial growth is controlled by the normal intestinal flora, but when a person is undergone to antibiotic treatment or when using gastric anti-acid drugs, the conditions become favorable for bacterial overgrowth. In addition to hospitals, *C. difficile* can be found in domestic animals, vegetables, meat and meat products, water, soil and fast foods (7–9).

This bacterium can be transmitted to humans through the fecal-oral route and from animals and animal products. Toxigenic *C. difficile* strains produces toxin A, toxin B and Binary Toxin (BT). Toxin A (*tcdA*) and toxin B (*tcdB*) genes are located close to each other on a Pathogencity Locus. Toxin A consists of 2710 amino acids (308 kDa) and toxin B consists of 2366 amino acids (270 kDa). These toxins are classified as lethal toxins among the group of large cytotoxic toxins (LCTS) (10) Toxin B causes more mucosal damage in colon comparing to toxin A, that’s why pathogenesis of strains lacking toxin B, is similar to non-toxigenic strains (11, 12).

There are not many reports about *C. difficile* infection in the Middle East, while the studies from rest of the world, such as Europe and North America show that infections caused by this bacterium are rapidly increasing (13).

According to the reports, ground meat can be a carrier for *C. difficile* and transmit the bacteria to humans. Moreover, there is little information about the prevalence of *C. difficile* in meat in Iran. Therefore, we analyzed the prevalence of *C. difficile* isolates and determined the prevalence of *cdd3, tcdA* and *tcdB* genes in beef samples in Kermanshah province.

## MATERIALS AND METHODS

### Specimens.

In this study, 100 random samples of ground beef were collected during March 2014 to March 2015. Samples were obtained from the butchers of Kermanshah and cities of West Islamabad, Sar-e Pol-e Zahab, Qasr-e Shirin and Dalahoo.

### Culture and Primary Identification.

One gram of ground meat samples was slowly mixed with ethanol 96% and incubated for 2 minutes at room temperature. The resulting solution was cultured on Cycloserine, Cefoxitin, Fructose Agar F (CCFA) (Hi Media -India) enriched with 5% sheep blood and 250 μg /ml cycloserine and 8 μg / ml Cefoxitin antibiotics. Cultured Plates were incubated at 37°C under anaerobic atmosphere for 5–7 days.

Suspected colonies with yellowish gray or white, round and slightly raised was identified with Gram staining.

### Molecular Methods.

DNA of confirmed colonies was extracted by a kit (Sinaclon) and presence of *tcdA, tcdB* and *cdd3* was investigated by PCR using specific primers (primers were designed based on sequences retrieved from Gen Bank). Isolates that were positive for *cdd3* gene in PCR were tested for *tcdA* and *tcdB*. List of used primers are shown in Table 1.

**Table 1.** Sequences of primers used for the amplification of *cdd3*, *tcdA*, and *tcdB* genes

**Primers**		**Sequence**	**Product bp**	**References**
*cdd3*	F	TGA TAA CTC TAG GTA ATA AGA CC	497	This study
	R	GTA ATC CAG ATA TGT TAG GTG G		
*tcdA*	F	GTG TAG ATT CAC TTT CCA ATG	404	This study
	R	TGA TAT TGA TGC TAA TCC TGG		
*tcdB*	F	GATGTTGATAATGTTGTGAGAG	653	This study
	R	TCA GCT ACT CCA CTT TCA TC		

A PCR reaction was performed in a total volume of 15 μl containing1X PCR buffer (Sinaclon, Iran), 0.2mM deoxynucleotide mix, 1.5 mM MgCl2, 0.6μM of primers, 1 unit of *Taq* DNA polymerase and 1 μl of DNA template. The program consisted of initial denaturation at 94°C for 5 minutes and 35 thermal cycles including denaturation at 94 for 30 seconds, annealing at 54°C for *cdd3, tcdA* and 53°C for *tcdB* genes, and a final extension at 72°C for 5 minutes. PCR products were isolated by 1% agarose gel electrophoresis and resulting bands were visualized by staining with Ethidium Bromide solution.

### Statistical Analysis.

Data were analyzed using the SPSS 16 software. The qualitative data were analyzed using chi-square test, and in all cases p < 0.05 was considered as significant.

## RESULTS

Suspected colonies were identified by Gram stain and the isolates were confirmed by molecular methods. From a total of 100 samples of ground beef, *C. difficile* was positive in 30 samples (30%).

**Fig. 1:**
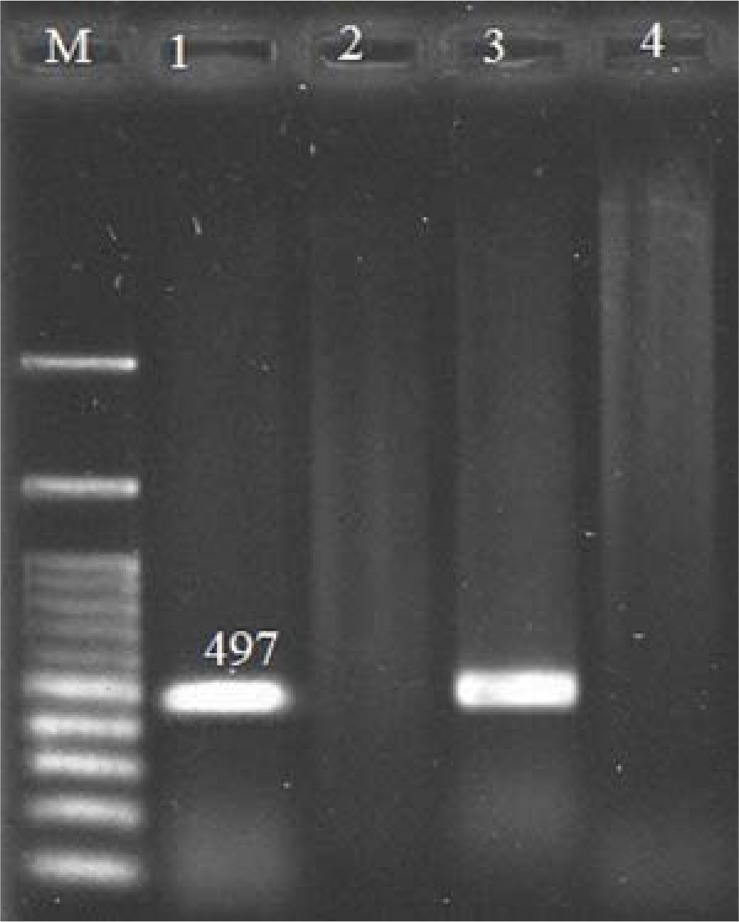
PCR Products Gel Electrophoresis for *cdd3* Gene. M:100bp Plus DNA Ladder RTU (Ready-to-Use), lane1: positive control, lane 2: *cdd3* Gene negative control, lane 3: *cdd3* gene positive sample, lane 4: *cdd3* gene negative sample.

**Fig. 2:**
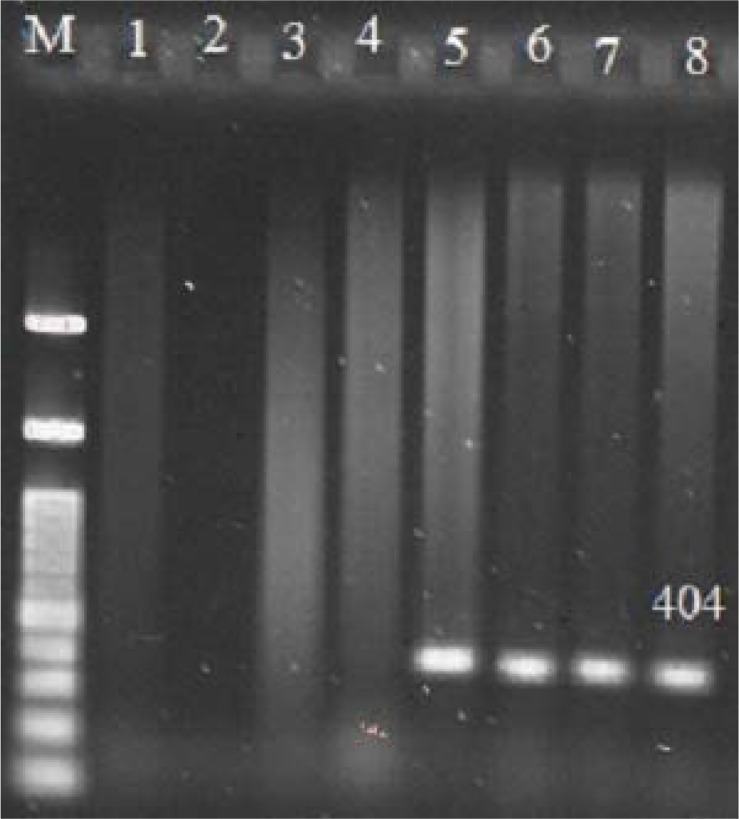
Agarose gel electrophoresis of PCR products for *tcdA* Gene. M:100bp Plus DNA Ladder RTU, lane1: positive control, lane 2, 3,4: *tcdA* Gene negative control, lane 5,6,7,8: *tcdA* gene positive sample.

**Fig. 3:**
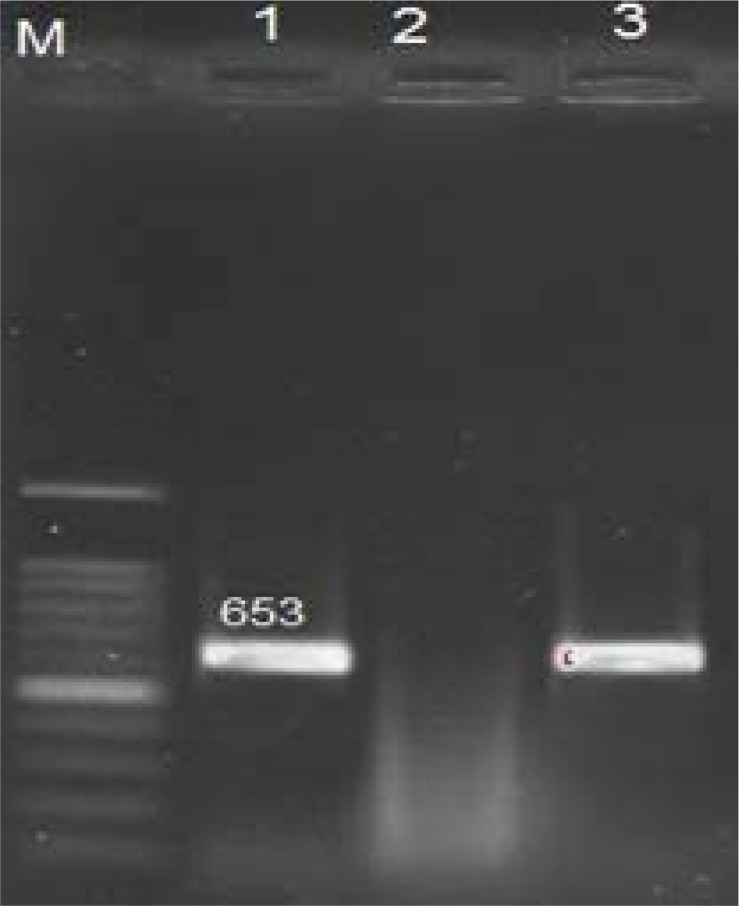
Agarose gel electrophoresis of PCR products for *tcdB* Gene. M: 100bp Plus DNA Ladder RTU, lane1: positive control, lane 2: *tcdB* Gene negative control, lane 3: *tcdB* gene positive sample.

The size of PCR products for *cdd3, tcdA* and *tcdB* genes were 497 bp (Fig. 1), 404 bp (Fig. 2) and 653 bp (Fig. 3), respectively. The frequency of *tcdA* and *tcdB* genes was 1% and 8%, respectively, while all the isolates harbored *cdd3* gene (30%). Among the 30 isolates, the combined dual frequency of, A^+^B^+^, A^−^B^+^ and A^+^B^−^ genes in *cdd3* positive *C. difficile* strains were 0%, 3.3%, 26.6%, respectively. Interestingly, 21 isolates (70%) were non-toxigenic (A^−^B) (Tables 1 and 2).

**Table 2.** The frequency of cultures, and *cdd3*, *tcdA* and *tcdB* genes in the collected samples obtained from Kermanshah province.

**Sample obtaining location**	**Culture**	***cdd3***	***tcdA***	***tcdB***
Kermanshah	31	13	1	0
West Islam Abad	47	16	7	1
Dlahoo	11	1	0	0
Qasre-Shirin	6	0	0	0
Sar-e Pol-e Zahab	5	0	0	0

## DISCUSSION

*C. difficile* is an important cause of nosocomial diarrhea. Spores of *C. difficile* are quite widespread in the environment and foods (14). It has been reported that the bacterium is a bovine pathogen which may be a serious threat to human health (15, 16). In various studies, the prevalence of infections with *C. difficile* has different values. As there are few studies concerning the prevalence of *C. difficile* in Iran, we aimed to assess the prevalence of the bacterium in ground meat samples in Kermanshah province.

Among samples of ground beef in several areas of Kermanshah Province, the frequency of A^+^B^+^, A^−^B^+^ and A^+^B^−^ were obtained 0, 3.3, and 26.6%, respectively. In this study, 21 (70%) non-toxigenic isolates were also isolated from minced meat samples which show that the rate of non-toxigenic strains is relatively high. It was believed that all toxigenic isolates of *C. difficile* produce both toxins (A^+^B^+^ strains) and the toxin B is capable of inducing CDAD without the presence of toxin A (17). But, later, severe cases of CDAD and pseudo-membranous colitis were observed that were arisen from the strains harboring toxin B and not having toxin A (strain A^−^B^+^).

Killgor reported that the frequency of *C. difficile* was 42% and also *C. difficile* were 42.2% 41.3% and 44.4% in beef, pork and turkey meat samples (18).

The prevalence of the bacteria in ground beef in studies from America and France was lower comparing to our data, even though in some studies no *C. difficile* was isolated from ground beef (19).

However, the prevalence of *C. difficile* in samples of ground beef in studies from America, Canada and the present study was 42%, 20.8 % and 30%, respectively. The different prevalence of *C. difficile* in ground beef samples in various studies can be attributed to the difference in sample size, geographical and regional conditions, and development of diagnostic test for *C. difficile* (18, 20).

There are some reports about the prevalence of toxigenic strains of *C.difficile* Iran. Rahimi et al reported a 1.65% frequency of *C. difficile* among 121 samples of beef, while the isolates produce both TcdA and TcdB toxins *tcdA* and *tcdB* genes (21). Moreover, in another study on chopped and ground meat carried out in Isfahan, the prevalence of *C. difficile* was 4% and 20% respectively which is close to our results. But with the difference that the number of samples examined in our study is much more than the study in Isfahan (22).

In study of Spain (2005), the prevalence of *tcd*A^+^B^+^ and *tcd*A^−^B^+^ encoding strains were 4.5%, 5% and 25.7%, 56.9% (23). The prevalence of A^−^B^+^isolates in Europe, China and Japan, was about 6.2%, 26.6% and 6.3% (24, 25). A^−^B^+^ strains are very rare and there are a few reports (26) that only one study that was conducted in one case of a total of 159 isolates. While in our study A^+^B^−^ strain was observed in 8 isolates (26.6%) (27). According to the aforementioned findings, we can conclude that most toxigenic isolates of the *C. difficile* in Iran are those variants. This could affect the expression of toxins and affect the clinical manifestation of the disease.

Based on the results of current study, there is a high rate of *C. difficile* of ground beef in Kermanshah province. According to studies in this field, ground meat can be a carrier for of *C. difficile* and transmit the bacteria to humans. Thus, control and supervision by the health organization and hygiene and sanitation and proper cooking can reduce food contamination with *C. difficile.* It seems that identifying virulence factors and the prevalence of these bacteria can be effective in the prevention and control of the disease (28, 29).

Furthermore, more studies must be done in different parts of the country to determine the prevalence of the *C. difficile* on food products would be helpful in future.
